# Protein-Protein Interaction Detection: Methods and Analysis

**DOI:** 10.1155/2014/147648

**Published:** 2014-02-17

**Authors:** V. Srinivasa Rao, K. Srinivas, G. N. Sujini, G. N. Sunand Kumar

**Affiliations:** ^1^Department of CSE, VR Siddhartha Engineering College, Vijayawada 520007, India; ^2^Department of CSE, Mahatma Gandhi Institute of Technology, Hyderabad 500075, India

## Abstract

Protein-protein interaction plays key role in predicting the protein function of target protein and drug ability of molecules. The majority of genes and proteins realize resulting phenotype functions as a set of interactions. The *in vitro* and *in vivo* methods like affinity purification, Y2H (yeast 2 hybrid), TAP (tandem affinity purification), and so forth have their own limitations like cost, time, and so forth, and the resultant data sets are noisy and have more false positives to annotate the function of drug molecules. Thus, *in silico* methods which include sequence-based approaches, structure-based approaches, chromosome proximity, gene fusion, *in silico* 2 hybrid, phylogenetic tree, phylogenetic profile, and gene expression-based approaches were developed. Elucidation of protein interaction networks also contributes greatly to the analysis of signal transduction pathways. Recent developments have also led to the construction of networks having all the protein-protein interactions using computational methods for signaling pathways and protein complex identification in specific diseases.

## 1. Introduction

Protein-protein interactions (PPIs) handle a wide range of biological processes, including cell-to-cell interactions and metabolic and developmental control [1]. Protein-protein interaction is becoming one of the major objectives of system biology. Noncovalent contacts between the residue side chains are the basis for protein folding, protein assembly, and PPI [2]. These contacts induce a variety of interactions and associations among the proteins. Based on their contrasting structural and functional characteristics, PPIs can be classified in several ways [3]. On the basis of their interaction surface, they may be homo- or heterooligomeric; as judged by their stability, they may be obligate or nonobligate; as measured by their persistence, they may be transient or permanent [4]. A given PPI may be a combination of these three specific pairs. The transient interactions would form signaling pathways while permanent interactions will form a stable protein complex.

Typically proteins hardly act as isolated species while performing their functions *in vivo* [5]. It has been revealed that over 80% of proteins do not operate alone but in complexes [6]. The substantial analysis of authenticated proteins reveals that the proteins involved in the same cellular processes are repeatedly found to be interacting with each other [7]. The study of PPIs is also important to infer the protein function within the cell. The functionality of unidentified proteins can be predicted on the evidence of their interaction with a protein, whose function is already revealed. The detailed study of PPIs has expedited the modeling of functional pathways to exemplify the molecular mechanisms of cellular processes [4]. Characterizing the interactions of proteins in a given proteome will be phenomenal to figure out the biochemistry of the cell [4]. The result of two or more proteins interacting with a definite functional objective can be established in several ways. The significant properties of PPIs have been marked by Phizicky and Fields [8].

PPIs can

- modify the kinetic properties of enzymes;
- act as a general mechanism to allow for substrate channeling;
- construct a new binding site for small effector molecules;
- inactivate or suppress a protein;
- change the specificity of a protein for its substrate through interaction with different binding partners;
- serve a regulatory role in either upstream or downstream level.

Uncovering protein-protein interaction information helps in the identification of drug targets [9]. Studies have shown that proteins with larger number of interactions (hubs) can include families of enzymes, transcription factors, and intrinsically disordered proteins, among others [10, 11]. However, PPIs involve more heterogeneous processes and the scope of their regulation is large. For more accurate understanding of their importance in the cell, one has to identify various interactions and determine the aftermath of the interactions [4].

In recent years, PPI data have been enhanced by guaranteed high-throughput experimental methods, such as two-hybrid systems, mass spectrometry, phage display, and protein chip technology [4]. Comprehensive PPI networks have been built from these experimental resources. However, the voluminous nature of PPI data is imposing a challenge to laboratory validation. Computational analysis of PPI networks is increasingly becoming a mandatory tool to understand the functions of unexplored proteins. At present, protein-protein interaction (PPI) is one of the key topics for the development and progress of modern system's biology.

## 2. Classification of PPI Detection Methods

Protein-protein interaction detection methods are categorically classified into three types, namely, *in vitro*, *in vivo*, and *in silico* methods. In *in vitro* techniques, a given procedure is performed in a controlled environment outside a living organism. The *in vitro* methods in PPI detection are tandem affinity purification, affinity chromatography, coimmunoprecipitation, protein arrays, protein fragment complementation, phage display, X-ray crystallography, and NMR spectroscopy. In *in vivo* techniques, a given procedure is performed on the whole living organism itself. The *in vivo* methods in PPI detection are yeast two-hybrid (Y2H, Y3H) and synthetic lethality. *In silico* techniques are performed on a computer (or) via computer simulation. The *in silico* methods in PPI detection are sequence-based approaches, structure-based approaches, chromosome proximity, gene fusion, *in silico* 2 hybrid, mirror tree, phylogenetic tree, and gene expression-based approaches. The diagrammatic classification was given in Table 1.

### 2.1. *In Vitro* Techniques to Predict Protein-Protein Interactions

TAP tagging was developed to study PPIs under the intrinsic conditions of the cell [12]. Gavin et al. first attempted the TAP-tagging method in a high-throughput manner to analyse the yeast interactome [13]. This method is based on the double tagging of the protein of interest on its chromosomal locus, followed by a two-step purification process [14]. Proteins that remain associated with the target protein can then be examined and identified through SDS-PAGE [15] followed by mass spectrometry analysis [15], thereby identifying the PPI collaborator of the original protein of interest. An important dominance of TAP-tagging is its ability to identify a wide variety of protein complexes and to test the activeness of monomeric or multimeric protein complexes that exist *in vivo* [14]. The TAP when used with mass spectroscopy (MS) will identify protein interactions and protein complexes.

The advantage of the affinity chromatography is that it is highly responsive, can even detect weakest interactions in proteins, and also tests all the sample proteins equally for interaction with the coupled protein in the column. However, false positive results also arise in the column due to high specificity among proteins, even though they do not get involved in the cellular system. Thus protein interaction studies cannot fully rely on affinity chromatography and hence require other methods in order to crosscheck and verify results obtained. The affinity chromatography can also be associated with SDS-PAGE technique and mass spectroscopy in order to generate a high-throughput data.

Coimmunoprecipitation confirms interactions using a whole cell extract where proteins are present in their native form in a complex mixture of cellular components that may be required for successful interactions. In addition, use of eukaryotic cells enables posttranslational modification which may be essential for interaction and which would not occur in prokaryotic expression systems.

Protein microarrays are rapidly becoming established as a powerful means to detect proteins, monitor their expression levels, and probe protein interactions and functions. A protein microarray is a piece of glass on which various molecules of protein have been affixed at separate locations in an ordered manner [16]. Protein microarrays have seen tremendous progress and interest at the moment and have become one of the active areas emerging in biotechnology. The objective behind protein microarray development is to achieve efficient and sensitive high-throughput protein analysis, carrying out large numbers of determinations in parallel by automated process.

Protein-fragment complementation assay is another method of proteomics for the identification of protein-protein interactions in biological systems. Protein-fragment complementation assays (PCAs) are a family of assays for detecting protein-protein interactions (PPIs) that have been introduced to provide simple and direct ways to study PPIs in any living cell, multicellular organism, or *in vitro* [17]. PCAs can be used to detect PPI between proteins of any molecular weight and expressed at their endogenous levels. The two choices for protein identification using a mass spectroscopy are peptide fingerprinting and shotgun proteomics [18]. For peptide fingerprinting, the eluted complex is separated using SDS-PAGE. The gel is either Coomassie-stained or silver-stained and bands unique to the test sample and hopefully containing a single protein are excised, enzymatically digested, and analyzed by mass spectrometry. The mass of these peptides is determined and matched to a peptide database to determine the source protein. The gel also provides a rough estimate of the molecular weight of the protein. Since only unique bands are cut out, background bands are not identified. Abundant background proteins may obscure target proteins while less abundant proteins may fall below the limits of detection by staining. This method works well with purified samples containing only a handful of proteins. Alternatively, for shotgun proteomics, the entire eluate, containing many proteins, is digested. Shotgun proteomics is currently the most powerful strategy for analyzing such complicated mixtures.

There are different implementations of the phage display methodology as well as different applications [19]. One of the most common approaches used is the M13 filamentous phage. The DNA encoding the protein of interest is ligated into the gene encoding one of the coat proteins of the virion. Normally, the process is followed by computational identification of potential interacting partners and a yeast two-hybrid validation step, but the method is a new born one [20].

X-ray crystallography [21] is essentially a form of very high resolution microscopy, which enables visualization of protein structures at the atomic level and enhances the understanding of protein function. Specifically it shows how proteins interact with other molecules and the conformational changes in case of enzymes. Armed with this information, we can also design novel drugs that target a particular target protein.

In the recent past, the researchers have shown interest in the analysis of protein-protein interaction by nuclear magnetic resonance (NMR) spectroscopy [21]. The location of binding interface is a crucial aspect in the protein interaction analysis. The basis for the NMR spectroscopy is that magnetically active nuclei oriented by a strong magnetic field absorb electromagnetic radiation at characteristic frequencies governed by their chemical environment [22, 23].

### 2.2. *In Vivo* Techniques to Predict Protein-Protein Interactions

Y2H method is an *in vivo* method applied to the detection of PPIs [24]. Two protein domains are required in the Y2H assay which will have two specific functions: (i) a DNA binding domain (DBD) that helps binding to DNA, and (ii) an activation domain (AD) responsible for activating transcription of DNA. Both domains are required for the transcription of a reporter gene [25]. Y2H analysis allows the direct recognition of PPI between protein pairs. However, the method may incur a large number of false positive interactions. On the other hand, many true interactions may not be traced using Y2H assay, leading to false negative results. In an Y2H assay, the interacting proteins must be localized to the nucleus, since proteins, which are less likely to be present in the nucleus are excluded because of their inability to activate reporter genes. Proteins, which need posttranslational modifications to carry out their functions, are unlikely to behave or interact normally in an Y2H experiment. Furthermore, if the proteins are not in their natural physiological environment, they may not fold properly to interact [26]. During the last decade, Y2H has been enriched by designing new yeast strains containing multiple reporter genes and new expression vectors to facilitate the transformation of yeast cells with hybrid proteins [27]. Other widely used techniques, such as bioluminescence resonance energy transfer (BRET), fluorescence resonance energy transfers (FRET), and bimolecular fluorescence complementation (BiFC), require extensive instrumentation. FRET uses time-correlated single-photon counting to predict protein interactions [28].

Synthetic lethality is an important type of *in vivo *genetic screening which tries to understand the mechanisms that allow phenotypic stability despite genetic variation, environmental changes, and random events such as mutations. This methodology produces mutations or deletions in two or more genes which are viable alone but cause lethality when combined together under certain conditions [29–33]. Compared with the results obtained in the aforesaid methods, the relationships detected by synthetic lethality do not require necessity of physical interaction between the proteins. Therefore, we refer to this type of relationships as functional interactions.

### 2.3. In Silico Methods for the Prediction of Protein-Protein Interactions

The yeast two-hybrid (Y2H) system and other *in vitro* and *in vivo* approaches resulted in large-scale development of useful tools for the detection of protein-protein interactions (PPIs) between specified proteins that may occur in different combinations. However, the data generated through these approaches may not be reliable because of nonavailability of possible PPIs. In order to understand the total context of potential interactions, it is better to develop approaches that predict the full range of possible interactions between proteins [4].

A variety of *in silico* methods have been developed to support the interactions that have been detected by experimental approach. The computational methods for *in silico* prediction include sequence-based approaches, structure-based approaches, chromosome proximity, gene fusion, *in silico *2 hybrid, mirror tree, phylogenetic tree, gene ontology, and gene expression-based approaches. The list of all webservers of *in silico* methods was given in Table 2.

#### 2.3.1. Structure-Based Prediction Approaches

The idea behind the structure-based method is to predict protein-protein interaction if two proteins have a similar structure. Therefore, if two proteins A and B can interact with each other, then there may be two other proteins A′ and B′ whose structures are similar to those of proteins A and B; then it is implied that proteins A′ and B′ can also interact with each other. But most proteins may not be having known structures; the first step for this method is to guess the structure of the protein based on its sequence. This can be done in different ways. The PDB database offers useful tools and information resources for researchers to build the structure for a query protein [34]. Using the multimeric threading approach, Lu et al. [35] have made 2,865 protein-protein interactions in yeast and 1,138 interactions have been confirmed in the DIP [36].

Recently, Hosur et al. [37] developed a new algorithm to infer protein-protein interactions using structure-based approach. The Coev2Net algorithm, which is a three-step process, involves prediction of the binding interface, evaluation of the compatibility of the interface with an interface coevolution based model, and evaluation of the confidence score for the interaction [37]. The algorithm when applied to binary protein interactions has boosted the performance of the algorithm over existing methods [38]. However, Zhang et al. [39] have used three-dimensional structural information to predict PPIs with an accuracy and coverage that are superior to predictions based on nonstructural evidence.

#### 2.3.2. Sequence-Based Prediction Approaches

Predictions of PPIs have been carried out by integrating evidence of known interactions with information regarding sequential homology. This approach is based on the concept that an interaction found in one species can be used to infer the interaction in other species. However, recently, Hosur et al. [37] developed a new algorithm to predict protein-protein interactions using threading-based approach which takes sequences as input. The algorithm, iWARP (Interface Weighted RAPtor), which predicts whether two proteins interact by combining a novel linear programming approach for interface alignment with a boosting classifier [37] for interaction prediction. Guilherme Valente et al. introduced a new method called Universal *In Silico* Predictor of Protein-Protein Interactions (UNISPPI), based on primary sequence information for classifying protein pairs as interacting or noninteracting proteins [40]. Kernel methods are hybrid methods which use a combination of properties like protein sequences, gene ontologies, and so forth [41]. However, there are two different methods under sequence-based criterion.

*(1) Ortholog-Based Approach.* The approach for sequence-based prediction is to transfer annotation from a functionally defined protein sequence to the target sequence based on the similarity. Annotation by similarity is based on the homologous nature of the query protein in the annotated protein databases using pairwise local sequence algorithm [42]. Several proteins from an organism under study may share significant similarities with proteins involved in complex formation in other organisms.

The prediction process starts with the comparison of a probe gene or protein with those annotated proteins in other species. If the probe gene or protein has high similarity to the sequence of a gene or protein with known function in another species, it is assumed that the probe gene or protein has either the same function or similar properties. Most subunits of protein complexes were annotated in that way. When the function is transferred from a characterized protein to an uncharacterized protein, ortholog and paralog concepts should be applied. Orthologs are the genes in different species that have evolved from a common ancestral gene by speciation. In contrast, paralogs usually refer to the genes related by duplication within a genome [43]. In broad sense, orthologs will retain the functionality during the course of evolution, whereas paralogs may acquire new functions. Therefore, if two proteins—A and B—interact with each other, then the orthologs of A and B in a new species are also likely to interact with each other.

*(2) Domain-Pairs-Based Approach. *A domain is a distinct, compact, and stable protein structural unit that folds independently of other such units. But most of times, domains are defined as distinct regions of protein sequence that are highly conserved in the process of evolution. As individual structural and functional units, protein domains play an important role in the development of protein structural class prediction, protein subcellular location prediction, membrane protein type prediction, and enzyme class and subclass prediction.

Conventionally, protein domains are used for basic research and also for structure-based drug designing. In addition, domains are directly involved in the intermolecular interaction and hence must be fundamental to protein-protein interaction. Multiple studies have shown that domain-domain interactions (DDIs) from different experiments are more consistent than their corresponding PPIs [44]. So, it is quite reliable to use the domains and their interactions for prediction of the protein-protein interactions and vice versa [45].

#### 2.3.3. Chromosome Proximity/Gene Neighbourhood

With the ever increasing number of the completely sequenced genomes, the global context of genes and proteins in the completed genomes has provided the researchers with the enriched information needed for the protein-protein interaction detection. It is well known that the functionally related proteins tend to be organized very closely into regions on the genomes in prokaryotes, such as operons, the clusters of functionally related genes transcribed as a single mRNA. If the neighborhood relationship is conserved across multiple genomes, then it will be more relevant for implying the potential possibility of the functional linkage among the proteins encoded by the related genes. And this evidence was applied to study the functional association of the corresponding proteins. This relationship was confirmed by the experimental results and shown to be more independent of relative gene orientation. Recently, it has been found that there is functional link among the adjacent bidirectional genes along the chromosome [46]. Interestingly, in most cases, the relationship among adjacent bidirectionally transcribed genes with conserved gene orientation is that one gene encodes a transcriptional regulator and the other belongs to nonregulatory protein [47]. It has been found that most of the regulators control the transcription of the diver gently transcribed target gene/operon and automatically regulate their own biosynthesis as well. This relationship provides another way to predict the target processes and regulatory features for transcriptional regulators. One of the pitfalls of this method is that it is directly suitable for bacterial genome since gene neighboring is conserved in the bacteria.

#### 2.3.4. Gene Fusion

Gene fusion, which is often called as Rosetta stone method, is based on the concept that some of the single-domain containing proteins in one organism can fuse to form a multidomain protein in other organisms [48, 49]. This domain fusion phenomenon indicates the functional association for those separate proteins, which are likely to form a protein complex. It has been shown that fusion events are particularly common in those proteins participating in the metabolic pathway [50, 51]. This method can be used to predict protein-protein interaction by using information of domain arrangements in different genomes. However, it can be applied only to those proteins in which the domain arrangement exists.

#### 2.3.5. In Silico Two-Hybrid (I2h)

The method is based on the assumption that interacting proteins should undergo coevolution in order to keep the protein function reliable. In other words, if some of the key amino acids in one protein changed, the related amino acids in the other protein which interacts with the mutated counter partner should also make the compulsory mutations as well. During the analysis phase, the common genomes containing those two proteins will be identified through multiple sequence alignments and a correlation coefficient will be calculated for every pair of residues in the same protein and between the proteins [52]. Accordingly, there are three different sets for the pairs: two from the intraprotein pairs and one from the interprotein pairs. The protein-protein interaction is inferred based on the difference from the distribution of correlation between the interacting partners and the individual proteins. Since I2h analysis is based on the prediction of physical closeness between residue pairs of the two individual proteins, the result from this method automatically indicates the possible physical interaction between the proteins.

#### 2.3.6. Phylogenetic Tree

Another important method for detection of interaction between the proteins is phylogenetic tree. The phylogenetic tree gives the evolution history of the protein. The mirror tree method predicts protein-protein interactions under the belief that the interacting proteins show similarity in molecular phylogenetic tree because of the coevolution through the interaction [53]. The underlying principle behind the method is that the coevolution between the interacting proteins can be reflected from the degree of similarity from the distance matrices of corresponding phylogenetic trees of the interacting proteins [54]. The set of organisms common to the two proteins are selected from the multiple sequence alignments (MSA) and the results are used to construct the corresponding distance matrix for each protein. The BLAST scores could also be used to fill the matrices. Then the linear correlation is calculated among these distance matrices. High correlation scores indicate the similarity between the phylogenetic trees and therefore the proteins are considered to have the interaction relationship. The MirrorTree method is used to detect the coevolution relationship between proteins and the results are used to infer the possibility of their physical interaction.

#### 2.3.7. Phylogenetic Profile

The notion for this method is that the functionally linked proteins tend to coexist during the evolution of an organism [55]. In other words, if two proteins have a functional linkage in a genome, there will be a strong pressure on them to be inherited together during evolution process [51]. Thus, their corresponding orthologs in other genome will be preserved or dropped. Therefore, we can detect the presence or absence (cooccurrence) of proteins in the phylogenetic profile. A phylogenetic profile describes an occurrence of a certain protein in a set of genomes: if two proteins share the same phylogenetic profiling, this indicates that the two proteins have the functional linkage. In order to construct the phylogenetic profile, a predetermined threshold of BLASTP *E*-value is used to detect the presence or absence of the homological proteins on the target genome with the source genomes. This method gives promising results in the detection of the functional linkage among the proteins and, at the same time, assigns the functions to query proteins. Even though the phylogenetic profile has shown great potential for building the functional linkage network on the full genome level, the following two pitfalls should be mentioned: one is that this method is based on full genome sequences and the other is that the functional linkage between proteins is detected by their phylogenetic profiling, so it is difficult to use the method for those essential proteins in the cell where no difference can be detected from the phylogenetic profile. Moreover, even though the increasing number in the source genome set can improve the prediction accuracy, there may be an upper limit for this method.

Many genomic events contribute to the noise during the coevolution, such as gene duplication or the possible loss of gene functions in the course of evolution, which could corrupt the phylogenetic profile of single genes. Phylogenetic-profile-based methods conceded satisfactory performance only on prokaryotes but not on eukaryotes [56].

#### 2.3.8. Gene Expression

The method takes the advantage of high-throughput detection of the whole gene transcription level in an organism. Gene expression means the quantification of the level at which a particular gene is expressed within a cell, tissue or organism under different experimental conditions and time intervals. By applying the clustering algorithms, different expression genes can be grouped together according to their expression levels, and the resultant gene expression under different experimental conditions can help to enunciate the functional relationships of the various genes. A lot of research has also been carried out to investigate the relationship between gene coexpression and protein interaction [57]. Based on the yeast expression data and proteome data, proteins from the genes belonging to the common expression-profiling clusters are more likely to interact with each other than proteins from the genes belonging to different clusters. In other studies, it has been confirmed that adjacent genes tend to be expressed both in the eukaryotes and prokaryotes. The gene coexpression concept is an indirect way to infer the protein interaction, suggesting that it may not be appropriate for accurate detection of protein interactions. However, as a complementary approach, gene coexpression can be used to validate interactions generated from other experimental methods.

## 3. Comparison of Protein-Protein Interaction Methods

Each of the above methods has been applied to detect the protein-protein interaction in both the prokaryotes and eukaryotes. The results show that most of them fit better for the prokaryotes than eukaryotes [14]. The significant increase for the coverage among those studies during the past several years could be mainly because of the increase in the number of genomes being decoded. This is because the more the number of genomes used in the study, the higher the coverage that the methods can reach. With the accumulation of fully sequenced genomes, the information content in the reference genome set is expected to increase. Accordingly, the prediction accuracy would increase with more genomes incorporated in the study. It can be anticipated that, with more and more genomes available in the future, the prediction potential will be improved and the corresponding combined methods will get higher coverage and accuracy. One thing that should be mentioned is that the selection of the standard used for the evaluation of the methods has a great impact on the coverage and accuracy. Besides the Operon and Swiss-Prot key word recovery used in the above studies, the KEGG has been used as the standard in Search Tool for the Retrieval of Interacting Genes (STRING) database [58]. It can be expected that the prediction coverage and accuracy will be different for each method under different standards. Obviously, the achieved highest coverage for the gene order method based on the operon standard indicates that the method is strongly related to operon.

Recent technological advances have allowed high-throughput measurements of protein-protein interactions in the cell, producing protein interaction networks for different species at a rapid pace. However, high-throughput methods like yeast two-hybrid, MS, and phage display have experienced high rates of noise and false positives. There are some verification methods to know the reliability of these high throughput interactions. They are Expression Profile Reliability (EPR index), Paralogous Verification Method (PVM) [59] Protein Localization Method (PLM) [60], and Interaction Generalities Measures IG1 [61] and IG2 [62]. EPR [63] method compares protein interaction with RNA expression profiles whereas PVM analyzes paralogs of interactors for comparison. The IG1 measure is based on the idea that interacting proteins that have no further interactions beyond level-1 are likely to be false positives. The IG2 measure uses the topology of interactions. Bayesian approaches have also been used for calculation of reliability [64–66]. The PLM gives the true positives (TP) as interacting proteins, which need to be localized in the same cellular compartment or annotated to have a common cellular role. So, in order to counter these errors, many methods have been developed which provides confidence scores with each interaction. Also, the methods that assign scores to individual interactions generally perform better than those with the set of interactions obtained from an experiment or a database [67].

## 4. Computational Analysis of PPI Networks

A PPI network can be described as a heterogeneous network of proteins joined by interactions as edges. The computational analysis of PPI networks begins with the illustration of the PPI network arrangement. The simplest sketch takes the form of a mathematical graph consisting of nodes and edges [68]. Protein is represented as a node in such a graph and the proteins that interact with it physically are represented as adjacent nodes connected by an edge. An examination of the network can yield a variety of results. For example, neighboring proteins in the graph probably may share more the same functionality. In addition to the functionality, densely connected subgraphs in the network are likely to form protein complexes as a unit in certain biological processes. Thus, the functionality of a protein can be inferred by spotting at the proteins with which it interacts and the protein complexes to which it resides. The topological prediction of new interactions is a novel and useful option based exclusively on the structural information provided by the PPI network (PPIN) topology [69]. Some algorithms like random layout algorithm, circular layout algorithm, hierarchical layout algorithm, and so forth are used to visualize the network for further analysis. Precisely, the computational analysis of PPI networks is challenging, with these major barriers being commonly confronted [4]:

1. the protein interactions are not stable;
2. one protein may have different roles to perform;
3. two proteins with distinct functions periodically interact with each other.

## 5. Role of PPI Networks in Proteomics

Predicting the protein functionality is one of the main objectives of the PPI network. Despite the recent comprehensive studies on yeast, there are still a number of functionally unclassified proteins in the yeast database which reflects the impending need to classify the proteins. The functional annotation of human proteins can provide a strong foundation for the complete understanding of cell mechanisms, information that is valuable for drug discovery and development [4]. The increased availability of PPI networks has developed various computational methods to predict protein functions. The availability of reliable information on protein interactions and their presence in physiological and pathophysiological processes are critical for the development of protein-protein-interaction-based therapeutics. The compendium of all known protein-protein interactions (PPIs) for a given cell or organism is called the interactome.

Protein functions may be predicted on the basis of modularization algorithms [4]. However, predictions found in this way may not be accurate because the accuracy of the modularization process itself is typically low. There are other methods which include the neighbor counting, Chi-square, Markov random field, Prodistin, and weighted-interactions-based method for the prediction of protein function [77]. For greater accuracy, protein functions should be predicted directly from the topology or connectivity of PPI networks [4]. Several topology-based approaches that predict protein function on the basis of PPI networks have been introduced. At the simplest level, the “neighbor counting method” predicts the function of an unexplored protein by the frequency of known functions of the immediate neighbor proteins [78]. The majority of functions of the immediate neighbors can be statistically assessed [79]. Recently, the number of common neighbors of the known protein and the unknown protein has been taken as the basis for the inference of function [80]. The weighted-graph-mining-based protein function prediction [81] is a novel approach in the area.

Protein-protein complex identification is the crucial step in finding the signal transduction pathways. Protein-protein complexes mostly consist of antibody-antigen and protease-inhibitor complexes [82]. Crystallography is the major tool for determining protein complexes at atomic resolution.

The complete analysis of PPIs can enable better understanding of cellular organization, processes, and functions. The other applications of PPI Network include biological indispensability analysis [4], assessing the drug ability of molecular targets from network topology [4], estimation of interactions reliability [83], identification of domain-domain interactions [84], prediction of protein interactions [85], detection of proteins involved in disease pathways [86], delineation of frequent interaction network motifs [87], comparison between model organisms and humans [88], and protein complex identification [89].

## 6. Protein Interaction Databases

The massive quantity of experimental PPI data being generated on steady basis has led to the construction of computer-readable biological databases in order to organize and to process this data. For example, the biomolecular interaction network database (BIND) is created on an extensible specification system that permits an elaborate description of the manner in which the PPI data was derived experimentally, often including links directly to the concluding evidence from the literature [75]. The database of interacting proteins (DIP) is another database of experimentally determined protein-protein binary interactions [36]. The biological general repository for interaction datasets (BioGRID) is a database that contains protein and genetic interactions among thirteen different species [70]. Interactions are regularly added through exhaustive curation of the primary literature to the databases. Interaction data is extracted from other databases including BIND and MIPS (Munich Information Center for protein se quences) [90], as well as directly from large-scale experiments [72]. HitPredict is a resource of high confidence protein-protein interactions from which we can get the total number of interactions in a species for a protein and can view all the interactions with confidence scores [71].

The Molecular Interaction (MINT) database is another database of experimentally derived PPI data extracted from the literature, with the added element of providing the weight of evidence for each interaction [72]. The Human Protein Interaction Database (HPID) was designed to provide human protein interaction information precomputed from existing structural and experimental data [91]. The information Hyperlinked over Proteins (iHOP) database can be searched to identify previously reported interactions in PubMed for a protein of interest [92]. IntAct [73] provides an open source database and toolkit for the storage, presentation, and analysis of protein interactions. The web interface provides both textual and graphical representations of protein interactions and allows exploring interaction networks in the context of the GO annotations of the interacting proteins. However, we have observed that the intersection and overlap between these source PPI databases is relatively small. Recently, the integration has been done and can be explored in the web server called APID (Agile Protein Interaction Data Analyzer) which is an interactive bioinformatics' web tool developed to allow exploration and analysis of currently known information about protein-protein interactions integrated and unified in a common and comparative platform [74]. The Protein Interaction Network Analysis (PINA2.0) platform is a comprehensive web resource, which includes a database of unified protein-protein interaction data integrated from six manually curated public databases and a set of built-in tools for network construction, filtering, analysis, and visualization [76]. The databases and number of interactions were tabled in Table 3.

## 7. Conclusion

While available methods are unable to predict interactions with 100% accuracy, computational methods will scale down the set of potential interactions to a subset of most likely interactions. These interactions will serve as a starting point for further lab experiments. The gene expression data and protein interaction data will improve the confidence of protein-protein interactions and the corresponding PPI network when used collectively. Recent developments have also led to the construction of networks having all the protein-protein interactions using computational methods for signal transduction pathways and protein complex identification in specific diseases.

## Figures and Tables

**Table 1:** Summary of PPI detection methods.

Approach	Technique	Summary
*In vitro *	Tandem affinity purification-mass spectroscopy (TAP-MS)	TAP-MS is based on the double tagging of the protein of interest on its chromosomal locus, followed by a two-step purification process and mass spectroscopic analysis
	Affinity chromatography	Affinity chromatography is highly responsive, can even detect weakest interactions in proteins, and also tests all the sample proteins equally for interaction
	Coimmunoprecipitation	Coimmunoprecipitation confirms interactions using a whole cell extract where proteins are present in their native form in a complex mixture of cellular components
	Protein microarrays (H)	Microarray-based analysis allows the simultaneous analysis of thousands of parameters within a single experiment
	Protein-fragment complementation	Protein-fragment complementation assays (PCAs) can be used to detect PPI between proteins of any molecular weight and expressed at their endogenous levels
	Phage display (H)	Phage-display approach originated in the incorporation of the protein and genetic components into a single phage particle
	X-ray crystallography	X-ray crystallography enables visualization of protein structures at the atomic level and enhances the understanding of protein interaction and function
	NMR spectroscopy	NMR spectroscopy can even detect weak protein-protein interactions

*In vivo *	Yeast 2 hybrid (Y2H) (H)	Yeast two-hybrid is typically carried out by screening a protein of interest against a random library of potential protein partners
	Synthetic lethality	Synthetic lethality is based on functional interactions rather than physical interaction

*In silico *	Ortholog-based sequence approach	Ortholog-based sequence approach based on the homologous nature of the query protein in the annotated protein databases using pairwise local sequence algorithm
	Domain-pairs-based sequence approach	Domain-pairs-based approach predicts protein interactions based on domain-domain interactions
	Structure-based approaches	Structure-based approaches predict protein-protein interaction if two proteins have a similar structure (primary, secondary, or tertiary)
	Gene neighborhood	If the gene neighborhood is conserved across multiple genomes, then there is a potential possibility of the functional linkage among the proteins encoded by the related genes
	Gene fusion	Gene fusion, which is often called as Rosetta stone method, is based on the concept that some of the single-domain containing proteins in one organism can fuse to form a multidomain protein in other organisms
	*In silico* 2 hybrid (I2H)	The I2H method is based on the assumption that interacting proteins should undergo coevolution in order to keep the protein function reliable
	Phylogenetic tree	The phylogenetic tree method predicts the protein-protein interaction based on the evolution history of the protein
	Phylogenetic profile	The phylogenetic profile predicts the interaction between two proteins if they share the same phylogenetic profile
	Gene expression	The gene expression predicts interaction based on the idea that proteins from the genes belonging to the common expression-profiling clusters are more likely to interact with each other than proteins from the genes belonging to different clusters

**Table 2:** The list of web servers with their references.

S. number	Web server	Function	Reference
1	Struct2Net	The Struct2Net server makes structure-based computational predictions of protein-protein interactions (PPIs)	http://groups.csail.mit.edu/cb/struct2net/webserver/
2	Coev2Net	Coev2Net is a general framework to predict, assess, and boost confidence in individual interactions inferred from a high-throughput experiment	http://groups.csail.mit.edu/cb/coev2net/
3	PRISM PROTOCOL	PRISM PROTOCOL is a collection of programs that can be used to predict protein-protein interactions using protein interfaces	http://prism.ccbb.ku.edu.tr/prism_protocol/
4	InterPreTS	InterPreTS uses tertiary structure to predict interactions	http://www.russell.embl.de/interprets
5	PrePPI	PrePPI predicts protein interactions using both structural and nonstructural information	http://bhapp.c2b2.columbia.edu/PrePPI/
6	iWARP	iWARP is a threading-based method to predict protein interaction from protein sequences	http://groups.csail.mit.edu/cb/iwrap/
7	PoiNet	PoiNet provides PPI filtering and network topology from different databases	http://poinet.bioinformatics.tw/
8	PreSPI	PreSPI predicts protein interactions using a combination of domains	http://code.google.com/p/prespi/
9	PIPE2	PIPE2 queries the protein interactions between two proteins based on specificity and sensitivity	http://cgmlab.carleton.ca/PIPE2
10	HomoMINT	HomoMINT predicts interaction in human based on ortholog information in model organisms	http://mint.bio.uniroma2.it/HomoMINT
11	SPPS	SPPS searches protein partners of a source protein in other species	http://mdl.shsmu.edu.cn/SPPS/
12	OrthoMCL-DB	OrthoMCL-DB is a graph-clustering algorithm designed to identify homologous proteins based on sequence similarity	http://orthomcl.org/orthomcl/
13	P-POD	P-POD provides an easy way to find and visualize the orthologs to a query sequence in the eukaryotes	http://ppod.princeton.edu/
14	COG	COG shows phylogenetic classification of proteins encoded in genomes	http://www.ncbi.nlm.nih.gov/COG/
15	BLASTO	BLASTO performs BLAST based on ortholog group data	http://oxytricha.princeton.edu/BlastO/
16	PHOG	PHOG web server identifies orthologs based on precomputed phylogenetic trees	http://phylogenomics.berkeley.edu/phog/
17	G-NEST	G-NEST is a gene neighborhood scoring tool to identify co-conserved, coexpressed genes	https://github.com/dglemay/G-NEST
18	InPrePPI	InPrePPI predicts protein interactions in prokaryotes based on genomic context	http://inpreppi.biosino.org/InPrePPI/index.jsp
19	STRING	STRING database includes protein interactions containing both physical and functional associations	http://string.embl.de
20	MirrorTree	The MirrorTree allows graphical and interactive study of the coevolution of two protein families and asseses their interactions in a taxonomic context	http://csbg.cnb.csic.es/mtserver/
21	TSEMA	TSEMA predicts the interaction between two families of proteins based on Monte Carlo approach	http://tsema.bioinfo.cnio.es/

**Table 3:** Protein interaction databases.

S. number	Database name	Total number of interactions (to date)	References	Source link	Number of species/organisms
1	BioGrid	7,17,604	[70]	http://thebiogrid.org/	60
2	DIP	76,570	[36]	http://dip.doe-mbi.ucla.edu/dip/Main.cgi	637
3	HitPredict	2,39,584	[71]	http://hintdb.hgc.jp/htp/	9
4	MINT	2,41,458	[72]	http://mint.bio.uniroma2.it/mint/	30
5	IntAct	4,33,135	[73]	http://www.ebi.ac.uk/intact/	8
6	APID	3,22,579	[74]	http://bioinfow.dep.usal.es/apid/index.htm	15
7	BIND	>3,00,000	[75]	http://bind.ca/	—
8	PINA2.0	3,00,155	[76]	http://cbg.garvan.unsw.edu.au/pina/	7
